# A review of malaria molecular markers for drug resistance in *Plasmodium falciparum* and *Plasmodium vivax* in China

**DOI:** 10.3389/fcimb.2023.1167220

**Published:** 2023-05-09

**Authors:** Siqi Wang, Fang Huang, He Yan, Jianhai Yin, Zhigui Xia

**Affiliations:** ^1^ National Institute of Parasitic Diseases, Chinese Center for Disease Control and Prevention, Shanghai, China; ^2^ National Center for International Research on Tropical Diseases, Shanghai, China; ^3^ National Health Commission (NHC) Key Laboratory of Parasite and Vector Biology (National Institute of Parasitic Diseases, Chinese Center for Disease Control and Prevention), Shanghai, China; ^4^ World Health Organization (WHO) Collaborating Centre for Tropical Diseases, National Center for International Research on Tropical Diseases, Shanghai, China; ^5^ Shanghai Municipal Center for Disease Control and Prevention, Shanghai, China

**Keywords:** *Plasmodium falciparum*, *Plasmodium vivax*, polymorphism, gene, China

## Abstract

China has now achieved the elimination of malaria, but it still faces severe challenges in the post-elimination stage. China continues to be plagued by imported malaria cases, and preventing re-transmission of imported malaria is critical. The effectiveness of antimalarial drugs for malaria control largely depends on the study of drug resistance markers *in vitro*. Monitoring molecular markers of parasite-associated drug resistance can help predict and manage drug resistance. There is currently a lack of systematic reviews of molecular markers for indigenous and imported malaria in China. Therefore, this review summarizes the published articles related to molecular marker polymorphism of indigenous and imported malaria cases in China in the past two decades, to study the mutation frequency and distribution of *crt*, *mdr1*, *dhps*, *dhfr* and *K13* gene resistance-related loci. This can provide a whole picture of molecular markers and the resistance mutations of imported cases in China, which has certain significance for drug resistance surveillance planning, safe and effective treatment, and preventing the recurrence of local transmission by imported malaria in China in the future.

## Introduction

Malaria is one of the most devastating infectious diseases. It imposes a severe burden on developing countries, especially in Southeast Asia and Africa. Of the five species of *Plasmodium* that infect humans, *Plasmodium falciparum* and *Plasmodium vivax* have the highest infection and morbidity rates. According to the 2022 World Health Organization (WHO) World Malaria Report, there are an estimated 247 million malaria cases globally in 2021, with an increase from 245 million in 2020 (WHO, 2022). The increase in malaria cases has been influenced by the disruption of services during the COVID-19 pandemic. Malaria deaths are estimated at 619,000 in 2021, a slight decrease compared to 2020 (WHO, 2022).

The widespread resistance of parasites to antimalarial drugs has attracted great attention in recent years. The emergence of parasite resistance has severely hindered the progress of malaria elimination. Resistance to chloroquine (CQ) in *P. falciparum* had emerged in Thailand as early as the late 1950s, and over time the drug was no longer effective (Payne, 1987). In the 1960s and 1970s, these resistant strains spread steadily through South America, Southeast Asia and India (Liu et al., 1995; Wellems and Plowe, 2001). Resistance also generally emerged in the late 1970s following the entry of sulfadoxine-pyrimethamine (SP) as an alternative treatment for *falciparum* malaria (Suebsaeng et al., 1986). Malaria treatment has been plagued by recurring resistance in parasites until the advent of artemisinin. Artemisinin-based combination therapies (ACTs) were introduced in the 1990s and are now used to treat uncomplicated malaria (Fairhurst and Dondorp, 2016). With the widespread use of ACTs in malaria-endemic countries and the increase in insecticide-treated bed nets, malaria morbidity and mortality have decreased significantly globally (Bhattarai et al., 2007). However, artemisinin resistance was first reported in western Cambodia in 2008, and the emergence of resistance resulted in lower parasite clearance rates (Noedl et al., 2008; Dondorp et al., 2009; Ashley et al., 2014). Since then, artemisinin-resistant parasites have spread rapidly across Southeast Asia (Phyo et al., 2012; Kyaw et al., 2013). Recent studies have confirmed the emergence of artemisinin partial resistance in several areas of Africa, notably in Rwanda, Guyana and Papua New Guinea (Maïga-Ascofaré and May, 2016; Uwimana et al., 2021). In the absence of effective antimalarial drugs, the emergence of artemisinin resistance in Africa is likely to lead to a repeat of high mortality rates and this needs to be addressed through resistance surveillance. At present, *P. falciparum* is resistant to almost all antimalarial drugs. Meanwhile, *P. vivax* also developed resistance to chloroquine and primaquine in Southeast Asia during the 1990s (Murphy et al., 1993; Looareesuwan et al., 1997).

China has also suffered from malaria since ancient times. After long-term unremitting struggle and efforts, China reported zero indigenous cases in 2017 (Feng et al., 2018). Although WHO certified China as a malaria-free country in 2021, China is still plagued by imported malaria cases (Liu, 2014; WHO, 2022). In the context of current antimalarial drug resistance globally, the threat of imported drug-resistant parasites must be monitored and addressed timely. The effectiveness of antimalarial drugs for malaria control relies heavily on the study of resistance markers *in vitro*, and monitoring parasite-associated molecular markers of resistance can help predict and manage drug resistance (Fairhurst and Dondorp, 2016; Haldar et al., 2018). This review summarizes the published articles related to molecular marker polymorphism of indigenous and imported malaria cases in China in the past two decades, to study the mutation frequency and distribution of resistance-related loci. This can provide better understanding of antimalarial drug resistance marker surveillance in China, which has certain significance for the surveillance and management of imported cases and preventing re-transmission in the future.

## Study selection method

**Figure 1:**
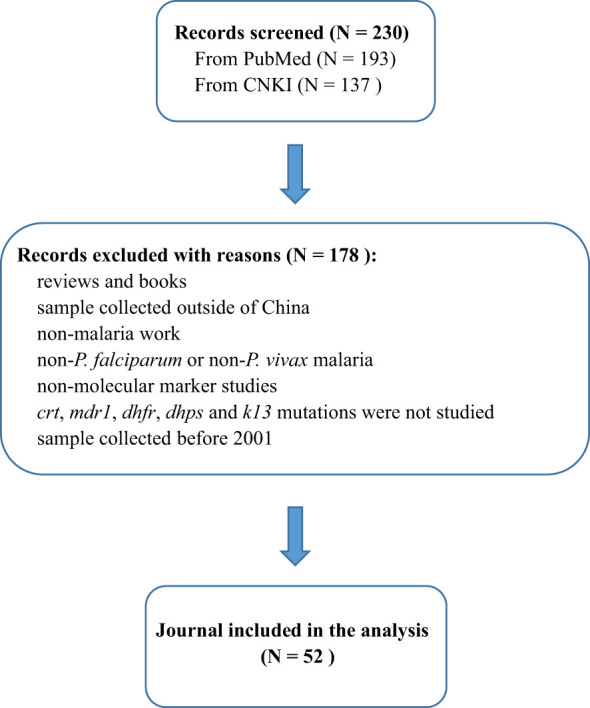
Flow diagram of study selection. The figure shows the number of manually screened and excluded publications identified in PubMed and CNKI, and finally screened the 52 publications counted in this study.

PubMed and CNKI databases were searched for peer-reviewed articles from China published between 1 January 2001 and 1 May 2022, that had genotyped the *crt*, *mdr1*, *dhfr*, *dhps* and *pfK13* genes of *P. falciparum* or *P. vivax* (**Figure 1**). The following search terms were used: “((malaria OR falciparum OR vivax) AND (molecular marker OR *crt* OR *mdr1* OR *dhfr* OR *dhps* OR *K13*)) AND (China OR China-Myanmar border) AND ((“2001/01/01” [Date - Publication]: “2022/05/01” [Date - Publication]))”. Studies related to parasites in the last two decades were screened and those based on transgenic parasites, books and modelling studies were excluded. In addition, the following information was extracted from the studies: the year samples were collected, study area, source of imported cases, sample size, and gene polymorphism.

## Results

### Literature screening results

According to the search conditions, a total of 230 literatures were obtained from PubMed and CNKI databases. Reviews and books, non-malaria studies and molecular marker studies were excluded. According to the abstract and results of the literature, studies containing non-*P. falciparum* or non-*P. vivax*, non-*crt* gene, non-*mdr1* gene, non-*dhfr* gene, non-*dhps* gene, non-*K13* gene and studies with samples collected before 2001 were all excluded. Finally, 52 literatures were included in this study (**Figure 1**). According to statistics, the total number of samples included in the study was 17,754, and its time span was from 2001 to 2019.

### The distribution of samples

**Figure 2:**
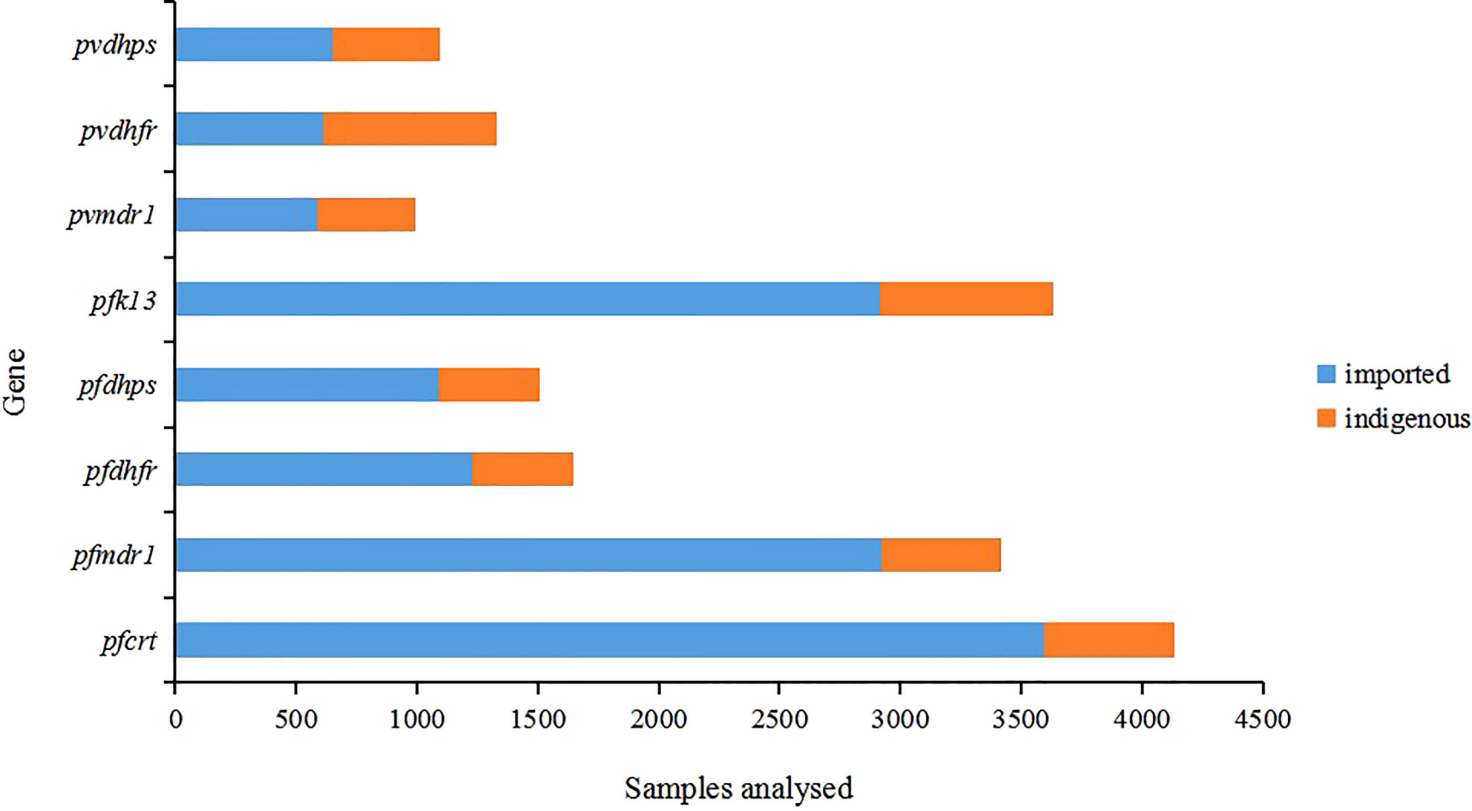
Distribution of samples by gene. All samples (n=17754) were from published studies (n=52). The indigenous cases were collected from 2001 to 2014 and the imported cases were collected from 2004 to 2019.

Among all samples, indigenous samples (76.7%) were collected from 2001 to 2014 and imported samples (23.3%) were collected from 2004 to 2019. The collection time of these samples was not continuous, and there were gaps in several years, which may be due to the time lag of sample research or the lack of research in this area. The proportion of imported samples was significantly higher than that of indigenous samples among different gene groups (*P* < 0.05) (**Figure 2**). For more than 63.5% of the 52 studies, the years of sample collection were from 2012 to 2019 (**Supplementary Tables 1–8**). More than 90% of the imported *P. falciparum* malaria samples in China were mainly imported from Africa, whereas all imported *P. vivax* cases in China came from Southeast Asia, especially Myanmar.

### Molecular marker of resistance

**Figure 3:**
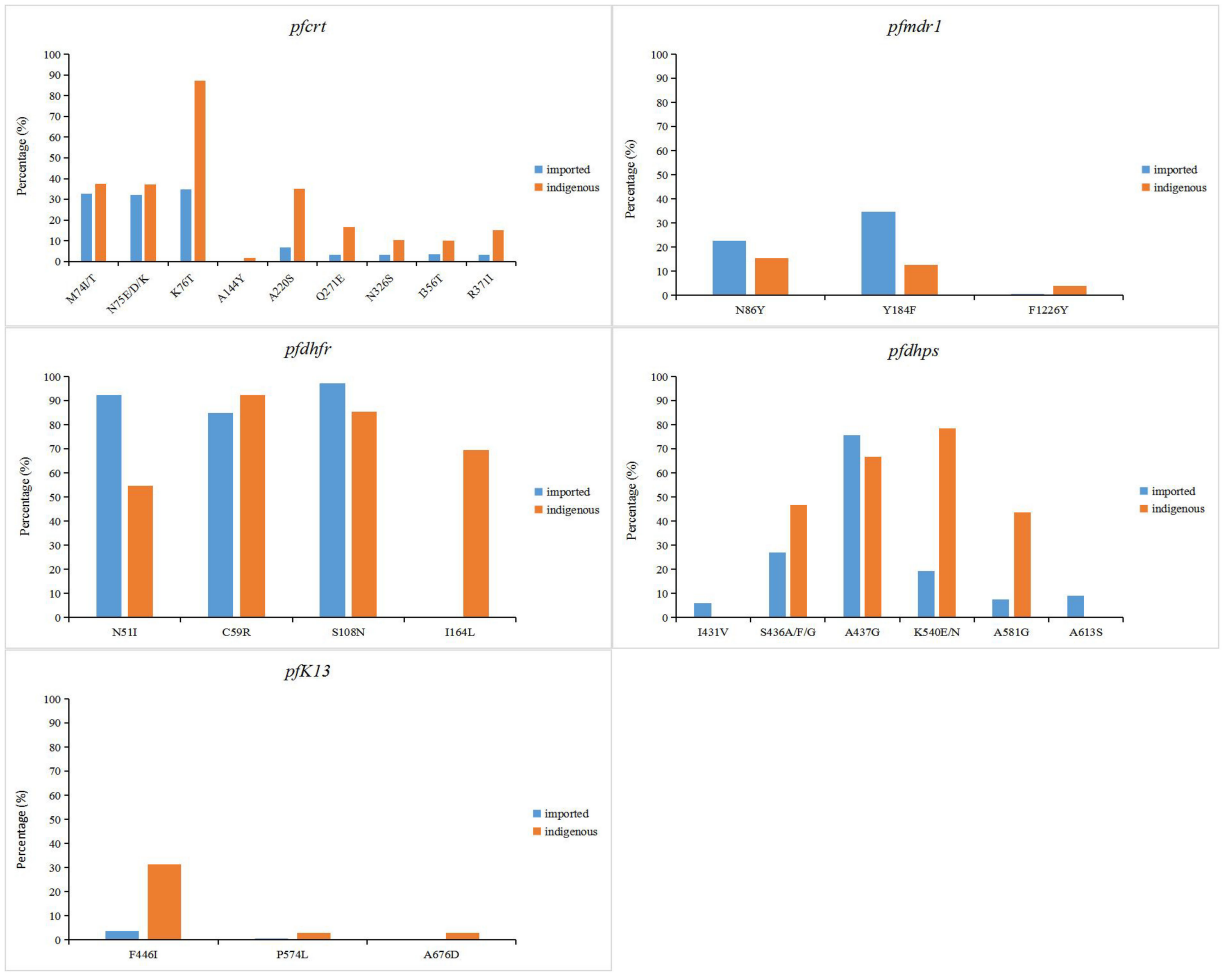
Point mutation frequencies of *pfcrt*, *pfmdr1*, *pfdhfr, pfdhps* and *pfK13* genes. The mutation sites with frequencies greater than 1% were shown in the figure. The indigenous cases of *P. falciparum* were collected from 2001 to 2014, while the imported cases were collected from 2007 to 2019.

In *P. falciparum* related resistance genes, the *pfcrt* gene of indigenous samples had the highest mutation frequency of K76T (87.1%), while M74I/T, N75E/D/K and A220S mutations were more common (37.5%, 37.3% and 35%, respectively) (**Figure 3**). Only K76T mutation was detected in samples from 2001 to 2007. In imported samples, mutations frequency at sites 74, 75 and 76 were similar (32.6%, 32.2% and 34.8%, respectively) and usually mutated simultaneously. In indigenous samples, the most common mutations of *pfmdr1* gene were N86Y and Y184F, accounting for 14% and 12.6%, respectively. The N1042D mutation of *pfmdr1* was detected in some samples, but the proportion was very low (only 0.8%) (**Supplementary Table 2**). Similar to the indigenous samples, the most common mutation of *pfmdr1* in the imported samples were N86Y and Y184F (22.7% and 34.7%, respectively) (**Figure 3**). The difference was that the mutation of N1042D was not detected in the imported samples, but the D1246Y mutation of *pfmdr1* was detected in the imported African samples, which was not detected in the indigenous samples. In indigenous samples, the mutations in N51I, C59R, S108N and I164L of *pfdhfr* were all over 50%, and C59R had the highest mutation frequency (92.4%). In imported samples, the mutation frequency of N51I, C59R and S108N in *pfdhfr* gene were high, accounting for 92.3%, 84.9% and 97.1%, respectively (**Figure 3**). The difference was that the mutation frequency of I164L in indigenous samples (69.5%) was significantly higher than that in imported samples (0.24%) (*P* < 0.05). For *pfdhps*, the highest mutation frequency was K540E/N (78.4%) in indigenous samples and A437G (75.7%) in imported samples. Mutations of I431V and A613S were detected in a small part of the imported samples. For *pfK13*, F446I had the highest mutation frequency, accounting for 31.4% of indigenous samples and 3.7% of imported samples (**Figure 3**).

**Figure 4:**
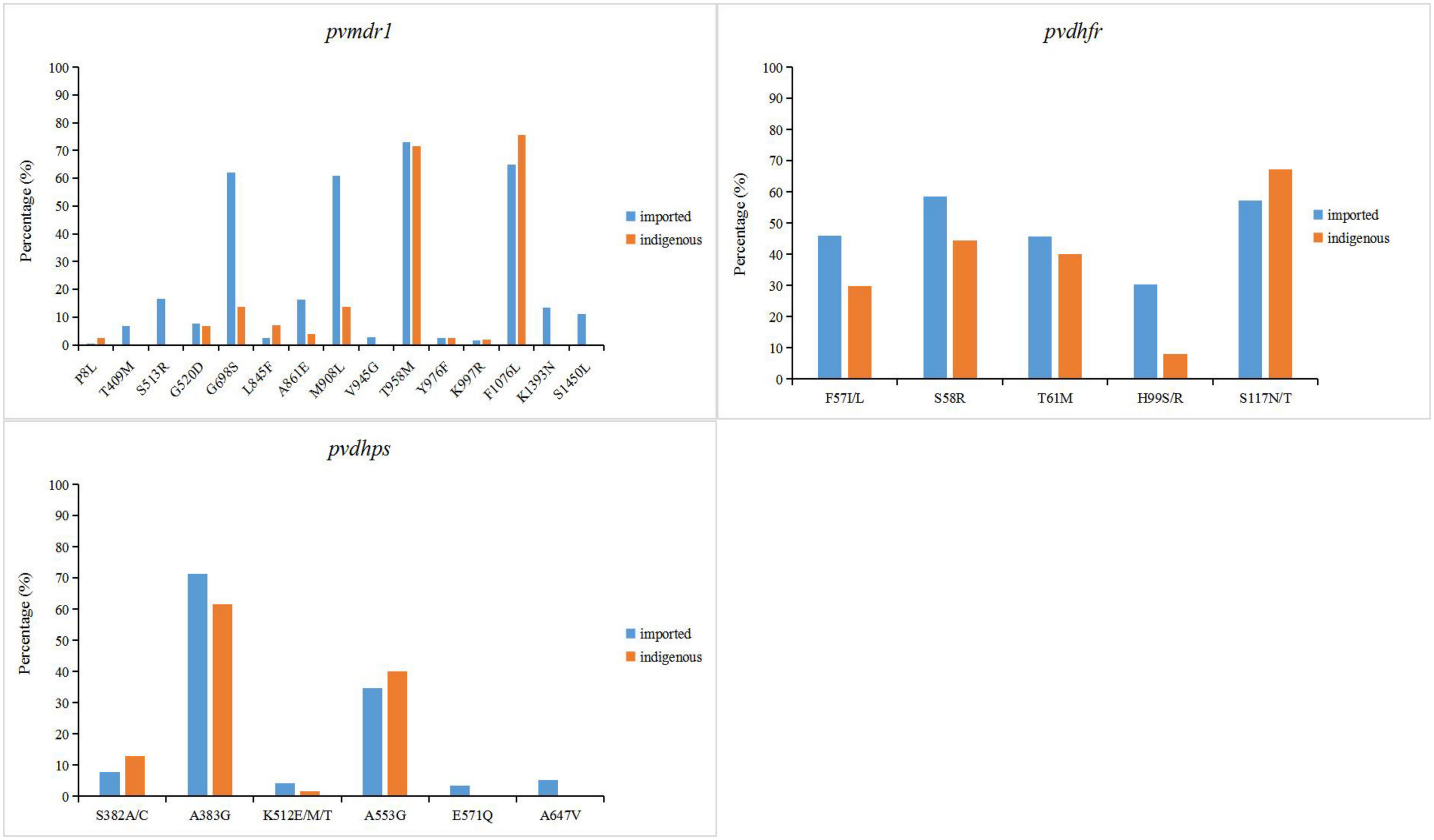
Point mutation frequencies of *pvmdr1*, *pvdhfr* and *pvdhps* genes. The mutation sites with frequencies greater than 1% were shown in the figure. The indigenous cases of *P. vivax* were collected from 2006 to 2012, while the imported cases were collected from 2004 to 2019.

In *P. vivax* related resistance genes, the mutation frequency of T958M and F1076L of *pvmdr1* gene was the highest in indigenous samples, which were 71.6% and 75.6%, respectively (**Figure 4**). Different from the indigenous samples, in addition to T958M (74.1%) and F1076L (64.9%), the mutation of *pvmdr1* gene was also higher at G698S (62%) and M908L (60.8%) in imported samples. In indigenous samples, the mutation frequency of S58R, T61M and S117N/T were high in *pvdhfr* gene, which were 44.4%, 40.1% and 67.3%, respectively, and the mutation frequency of A383G site in *pvdhps* gene was the highest (61.5%) (**Figure 4**). The highest mutation frequency of *pvdhfr* gene in imported samples was S58R (58.6%) and the mutation rate of A383G site in *pvdhps* gene was the highest (71.3%).

Based on the collected results, the period from 2001 to 2012 was defined as the early stage of the study, and the period from 2012 to 2019 as the late stage. In indigenous samples, *pfcrt*, *pfmdr1*, *pfdhfr*, *pfdhps*, *pvdhfr*, and *pvdhps* genes in the early stage were not significantly different from those in the late stage (*P >*0.05). Different from the late stage, mutations at N11Y, K189T, R225K, E252Q, I352T and P441L were newly found in less than five samples, but F446I was still the main mutation in *pfk13*. In the *pvmdr1* gene, mutations at P8L, G520D, G698S, L845F, A861E and M908L were newly discovered in the early stage, which were not found in the late stage. In the imported malaria samples, the mutation of each gene in the early stage showed no significant trend than the later stage (*P >*0.05).

## Discussion and conclusion

The study of genetic molecular markers of drug resistance can provide a better understanding of drug action and drug resistance mechanisms that are critical to achieving malaria treatment and control of transmission. The mutation of *P. falciparum* chloroquine resistance transporter gene (*pfcrt*) K76T has been confirmed to be closely related to CQ resistance, and those *P. falciparum* carrying the K76T mutation are generally accompanied by M74I, N75E and A220S mutations (Babiker et al., 2001; Djimdé et al., 2001; Sa and Twu, 2010). This phenomenon was also shown in the collection of indigenous and imported malaria samples in China, with a trend toward higher mutation frequency at sites 74-76 of *pfcrt* gene (**Figure 3**). In addition, mutations at loci Q271E, N326S, I356T, and R371I were found in which reported in the border area of Yingjiang County in Yunnan Province and Kachin area in Myanmar, which may be due to the frequent migration of populations in border areas to spread parasite drug resistance (Yang et al., 2007; Carrara et al., 2013; Wang et al., 2020; Si et al., 2021). *P. falciparum* multidrug resistance 1 (*pfmdr1*) genes are associated with resistance to CQ, mefloquine, quinine and artemisinin in *P. falciparum*, especially the N86Y allele (Sidhu et al., 2006; Feng et al., 2015; Gil and Krishna, 2017; Muiruri et al., 2018). From the data collected, mutations at the N86Y and Y184F sites of *pfmdr1* were common, mutations in Y184F and N86Y occurred simultaneously in most samples. (**Figure 3**). The F1226Y mutation was detected in only a few samples, studies confirmed that parasites with the F1226Y mutation increases parasite resistance to quinine and lumefantrine (Wang et al., 2020). Notably, the N1042D mutation was detected in the indigenous samples and studies have found that parasites with the N1042D mutation have increased susceptibility to pyronaridine (Bai et al., 2018), but the D1246Y mutation was detected in the imported African samples, which indicated the geographical differences in the production of malaria resistance (Xu et al., 2018; Zhou et al., 2019; She et al., 2020; Huang et al., 2021; Zhao et al., 2021).Mutations in dihydrofolate reductase (*dhfr*) and dihydropteroate synthase (*dhps*) are associated with parasite resistance to SP drugs (Triglia et al., 1997). The study found that mutations at N51I, C59R, S108N, and I164L of *pfdhfr* gene were associated with pyrimethamine resistance (Triglia et al., 1997; Happi et al., 2005). And mutations at S436A, A437G, K540E, A581G and A613S of *pfdhp* gene were associated with sulfadoxine resistance (Kublin et al., 2002; Berglez et al., 2004; Pearce et al., 2009). The mutation frequency of these sites of *pfdhfr* and *pfdhp*, which we collected, was high. This phenomenon is similar to studies in Myanmar, Thailand, and Cambodia in Southeast Asia (Anderson et al., 2005; Khim et al., 2005; Huang et al., 2012). It is estimated that *P. vivax*, which is endemic in Southeast Asia, has shown resistance to SP (**Figure 3**). Different from the indigenous samples imported samples of *falciparum* malaria from Africa have high mutation frequency (>80%) in N51I, C59R, and S108N of *pfdhfr*, and have lower mutation frequency at I164L (**Figure 3**). It can indicate that indigenous parasites may have developed high pyrimidine resistance. Therefore, close monitoring of the associated resistance to imported malaria in Africa is critical. The kelch13 (*K13*) propeller domain of *P. falciparum* has been shown to be associated with artemisinin resistance (Cheeseman et al., 2012; Miotto et al., 2013). It has been confirmed that the F446I, N458Y, M476I, Y493H, R539T, I543T, P553L, R561H and C580Y site mutations of the *pfK13* gene are closely related to artemisinin resistance (Huang et al., 2015; WHO, 2018; Feng et al., 2019). The F446I mutation of the *pfK13* gene was found to be predominant in the border areas of Myanmar and Yunnan Province of China (**Supplementary Table 8**).And the mutations of A578S and P574L loci of *pfK13* were more common in African imported samples (**Supplementary Table 8**). Although zero indigenous cases have been reported in China since 2017, cross-border malaria transmission due to *Anopheles* mosquitoes in the China-Myanmar border area and human importation from malaria-endemic areas in Southeast Asia and Africa make malaria management and drug resistance monitoring difficult (Feng et al., 2019).

.The proportion of imported *vivax* malaria cases has increased since the absence of indigenous cases in China, and there is still a risk of transmission (Zhang et al., 2022). *P. vivax* is the geographically most widespread malaria parasite and imposes a severe burden on global public health (Mueller et al., 2009; Howes et al., 2016). From the data collected, imported samples of *vivax* malaria in recent years came from Southeast Asia, especially Myanmar, where the malaria burden is the heaviest (**Supplementary Tables 5–7**). China’s Yunnan Province borders Myanmar, so there is a high risk of cross-border transmission of mosquito vectors, which still requires high attention. Due to the limitation of *in vitro* culture of *P. vivax*, the research of drug resistance molecular markers is full of challenges, so the research mainly focuses on the homologous molecular markers with *P. falciparum* (Wang et al., 2022). *P. vivax* multidrug resistance gene (*pvmdr1*), homologous to *pfmdr1*, has been identified as a possible genetic molecular marker of CQ resistance (Brega et al., 2005; Suwanarusk et al., 2007). The frequency of M908L (41.8%) or T958M (72.5%) mutations in the *pvmdr1* gene was higher in imported *vivax* samples of China (**Figure 4**). It is worth noting that mutations at the M908L, T958M, Y976F and F1076L sites have been confirmed to be closely related to CQ resistance (Brega et al., 2005; Chehuan et al., 2013; Li et al., 2020). Interestingly, the mutation frequencies of Y976F (2.4%) and F1076L (69.2%) in the China-Myanmar border area of imported *P. vivax* samples were significantly different (*P*<0.05) (**Figure 4**). This finding is consistent with studies in the Myanmar region, where Y976F is also rare (Nyunt et al., 2017), but these results are quite different from studies in the Thai-Cambodian border region, where the incidence of Y976F is high (Tantiamornkul et al., 2018). The resistance of *P. vivax* to SP has been shown to be caused by mutations in two genes, dihydrofolate reductase (*pvdhfr*) and dihydropteroate synthase (*pvdhps*) (Tjitra et al., 2002; Korsinczky et al., 2004). Among them, the mutations of *pvdhfr* codons 57, 58, 117, 173 and *pvdhps* codons 382, 383 and 553 were found to be associated with pyrimethamine and sulfadoxine resistance (Imwong et al., 2001; Imwong et al., 2003; Korsinczky et al., 2004; Huang et al., 2022). The *pvdhfr* gene with 57 site (36.3%) mutation mainly occurred in the border area between China and Myanmar, which was an imported case from Myanmar. The mutation at locus 117 of *pvdhfr* gene mainly occurred in the Myanmar, and the point mutation frequency of loci 58 and 117 were high, which were 51.0% and 62.7%, respectively (**Figure 4**). Almost all of the collected data carried at least one of the two mutations, 383 and 553, indicating that almost all of these parasites had developed resistance to sulfadoxine (**Figure 4**).

This study found that there was a lack of sample collection and testing in some years, which may be due to fewer studies in this period or delayed reporting. Most of the study period was from 2012 to 2019. Therefore, no significant trend in mutation sites of each gene has been observed over time. In addition, it was found that some mutation sites (especially *pfK13* gene) were detected in a small number of cases of these genes studied in this study. Due to the small number of detected mutations, it is necessary to continue to detect whether they have an impact on drug resistance in the future. At present, China has been eliminated malaria, but the risk of imported malaria continues to exist globally, especially resistant malaria in Southeast Asia and Africa. Therefore, surveillance for susceptibility of imported malaria to commonly used antimalarial drugs in China (including increased monitoring of the piperaquine resistance gene related gene *pfpm2-3*) should be integrated into the routine case surveillance in all provinces, which can provide complete evidence and address the risk of resistance to antimalarial drugs in time to ensure that treatment effects to prevent malaria retransmission.

## Author contributions

SW collected and analyzed the data. ZX revised the manuscript. All authors contributed to the article and approved the submitted version.
